# Assessment of Microbiological Contamination and Prevalence of Pathogenic Strains in Cattle Carcasses from Romanian Slaughterhouses

**DOI:** 10.3390/pathogens14030248

**Published:** 2025-03-03

**Authors:** Dariana-Olivia Brătfelan, Alexandra Tăbăran, Sorin Daniel Dan, Alexandru-Flaviu Tăbăran, Rodica Mărgăoan, Oana Lucia Crişan-Reget, Marian Mihaiu

**Affiliations:** Department of Animal Husbandry and Public Health, Faculty of Veterinary Medicine Cluj-Napoca, University of Agricultural Sciences and Veterinary Medicine Cluj, 400372 Cluj-Napoca, Romania; dariana-olivia.bratfelan@usamvcluj.ro (D.-O.B.); sorindan@usamvcluj.ro (S.D.D.); flaviu.tabaran@usamvcluj.ro (A.-F.T.); rodica.margaoan@usamvcluj.ro (R.M.); oana.reget@usamvcluj.ro (O.L.C.-R.); marian.mihaiu@usamvcluj.ro (M.M.)

**Keywords:** bovine slaughterhouses, pathogen detection, cross-contamination, meat safety

## Abstract

Food safety, particularly within the meat industry, is a significant concern addressed under the One Health concept, emphasizing the necessity of enhanced surveillance and hygiene protocols to mitigate contamination risks. This study assessed microbiological risks in Romanian bovine slaughterhouses by analyzing 150 samples from stool and carcasses at the post-evisceration and cooling stages over seven months in two abattoirs, using standardized microbiological methods and PCR to quantify aerobic colony counts (ACCs), *Enterobacteriaceae*, and pathogens (*E. coli*, *Salmonella* spp., and *Listeria* spp.). ACCs and *Enterobacteriaceae* levels decreased significantly [*p* < 0.05] during processing, highlighting effective hygiene measures. Pathogenic *E. coli* was identified in 14% of fecal samples and 5% of carcasses, indicating cross-contamination risks. *Salmonella* spp. were found in 28% of fecal samples but absent on carcasses, suggesting successful containment. *Listeria* spp. were rare and not detected on carcasses. PCR confirmed the presence of pathogenic strains in stool samples, emphasizing the need for strict hygiene practices and regular monitoring to improve meat safety and protect public health. In conclusion, the prevalence of *E. coli*, particularly serogroups like O101 and O26, and the absence of *Salmonella* and *Listeria* in carcass samples reflect both regional differences in pathogenic strains and the need for comprehensive, multi-stage control measures. Further studies should broaden pathogen surveillance to include more *E. coli* serogroups and implement stricter hygiene protocols to prevent cross-contamination during evisceration, skinning, and cooling. Regular monitoring of *Salmonella* and *Listeria*, especially in silage-fed cattle regions, along with improved coordination across the food production, health, and environmental sectors, is essential to mitigate contamination risks and safeguard public health.

## 1. Introduction

Food safety is a major issue and is extensively discussed within the framework of the One Health concept [1]. The idea of more intense surveillance of everything originating from animals and the risks associated with these is increasingly accepted. In order to ensure safety, additional measures beyond traditional post-mortem inspections are necessary in some situations, especially for animals raised for the meat industry, which are more likely to carry pathogenic bacteria that are important for food safety [2,3]. Contamination with such bacteria can occur through direct or indirect methods related to fecal contamination, which is most often encountered when hygienic conditions are not followed during slaughter [4]. Therefore, compliance with all hygiene programs (GHP and GMP), preventive measures, and the implementation of the HACCP system are essential [5]. Strict adherence to all correct slaughter practices is crucial to ensuring both public protection and meat quality [6]. Within the European Union, the use of current legislation related to meat hygiene (Reg. (EC) No. 852/2004 and Reg. (EC) No. 853/2004) provides a mandatory legal framework that is uniform for all operators [7,8]. To assess the risk and approximate the necessary measures, analyzing the slaughter process and how it is carried out is of central importance [9]. Specific data on the microbial load of carcasses during slaughter are essential for conducting an appropriate risk analysis [10,11], because carcasses may be contaminated even if this is not visible during post-mortem visual inspection [12]. To verify hygiene conditions during processing, the total number of aerobic mesophilic microorganisms and the total number of Enterobacteriaceae are monitored at the end of the slaughter line [13,14]. In the EU, Reg. (EC) No. 2073/2005 and Reg. (EC) No. 1441/2007 set microbiological criteria for carcasses at the end of the slaughter line [15,16].

Several studies have focused on meat safety knowledge and practices [17,18], while others have assessed the meat handling practices throughout the beef supply chain [19,20] and the bacteriological quality of meat in abattoirs and butcher shops across different countries [21,22]. Despite these studies, there is a significant gap in the literature regarding the investigation of food handlers’ daily practices and the potential sources of microbiological contaminants that could compromise meat quality [23].

Cattle are one of the main sources of red meat worldwide, including in Romania, where, due to the outbreak of African swine fever that affected the pig population, there has been an increase in cattle slaughter [24,25]. However, this species also faces a series of issues caused by viral, bacterial, parasitic, and protozoan diseases specific to cattle, which are of both economic and public health importance [26]. Beef production is one of the most important sources of livelihood for rural families, especially in developing countries, and the condemnation of carcasses or specific organs leads to significant economic losses for farmers and the livestock sector [27,28]. In the scientific literature, there are already a number of studies discussing the sources of and potential control measures for contamination with pathogenic bacteria at different stages of production: primary production [29,30,31], transport to slaughterhouse [6,32], slaughtering [33], carcass processing [34], distribution [35], and thermal treatment [36]; however, integrated studies that reveal the prevalence and potential risk factors associated with contamination with pathogenic bacteria across the entire production chain are limited, likely due to the complexity of this production system and the large number of samples that must be analyzed for a correct and accurate assessment.

Contamination of meat with pathogenic bacteria can occur at any stage of production [37]. Moreover, the prevention or elimination of associated risks can be carried out at stages other than the one where the risk arose [38]. Therefore, the integrated approach in this study constitutes an important basis for controlling the sources of bacterial contamination of meat destined for public consumption. The main goal of this research was to evaluate the prevalence of pathogenic bacteria associated with beef through studies conducted at two stages of production within the bovine slaughterhouse. The main directions addressed in the evaluation of microbiological risk factors were focused on the aerobic colony counts (ACCs) and Enterobacteriaceae number at the end of the production line and presence of pathogenic bacteria with a higher propensity to contaminate meat, such as *Salmonella*, pathogenic *E. coli*, and *Listeria*. The risk associated with the presence of these pathogens in beef has been reported in several studies in the literature [39,40,41,42,43,44], but their prevalence within the beef production chain in Romania is limited.

## 2. Materials and Methods

### 2.1. Sample Collection and Preparation

This study was conducted over seven months, from January to July 2023, at two Romanian abattoirs, each with an annual slaughter capacity exceeding 10 million kg. Abattoir A processed cattle, sheep, and pigs, with a capacity of up to 10 cattle carcasses per hour, averaging 45 cattle carcasses daily. Abattoir B, which exclusively processed cattle, had a higher capacity of up to 35 cattle carcasses per hour, averaging 90 per day. A total of 150 samples were collected for microbiological analysis, including 50 fecal samples (42 from Abattoir A and 8 from Abattoir B) via rectal swabbing, and 100 carcass samples taken at two stages of processing: 50 post-evisceration and 50 poSPSSst-cooling. For fecal samples, swabs were placed in sterile tubes, while for carcass samples, a moistened swab (0.85% saline solution) was used to rub a 400 cm^2^ area on the thoracic and abdominal regions, followed by a dry swab. These areas were selected due to the potential for contamination during evisceration. All samples were kept chilled during transport to the laboratory, where microbiological analyses were performed within 24 h of collection.

### 2.2. Aerobic Colony Counts (ACCs) and Enterobacteriaceae

Both swabs from each sampling carcass were homogenized for 60 s in 20 mL of 0.85% saline solution using a Stomacher machine. The resulting suspensions were plated using a spiral plater (EasySpiral, Interscience, Paris, France) on plate count agar (Oxoid AG, Hamburg, Germany) for ACCs and on violet-red bile glucose (VRBG) agar (ThermoF, Rockford, IL, USA) for Enterobacteriaceae. Agar plates were incubated following ISO 4833-1:2013 [45] (for plate count agar) and ISO 21528-2:2017 [46] (for VRBG agar) guidelines. Counts were expressed as CFU/cm^2^, with a detection limit of 4 CFU/cm^2^ for carcass samples.

### 2.3. Bacterial Isolation Methods

#### 2.3.1. *E. coli* Isolation Protocol

For *E. coli* isolation, samples were transferred in the laboratory into separate tubes, each containing 2 mL of nutrient broth, and incubated at 37 °C for 24 h, following the protocol described in ISO 16654:2001 [47]. Colonies capable of lactose fermentation were confirmed with an indole test, performed by adding Kovacs reagent to 2 mL of peptone water previously inoculated with 5 mL of enrichment medium. A positive indole test was indicated by a pink-red color change within 15 min. *E. coli* serotyping was based on the O antigen (somatic lipopolysaccharide), determined via sero-agglutination in wells with monovalent anticolibacillary sera from O1 to O157, using lactose-positive strains.

#### 2.3.2. *Salmonella* spp. Isolation Protocol

The identification method for *Salmonella* species followed the horizontal detection protocol for *Salmonella* spp. [48]. *Salmonella* isolation and identification required four successive steps. Non-selective pre-enrichment was performed by inoculating a 25 g sample into a non-selective liquid medium (buffered peptone water) and incubating at 35–37 °C for 16–20 h. The 25 g sample was homogenized with 225 mL of buffered peptone water at room temperature to obtain an initial dilution of 10^−1^, from which serial dilutions (10^−2^ to 10^−6^) were made, followed by incubation at 37 °C for 18 h. Selective enrichment in liquid media involved inoculating two different selective liquid media with the pre-enriched culture and incubating in parallel at different temperatures depending on the medium. A 0.1 mL and a 1 mL aliquot of pre-enriched culture were transferred to 10 mL Rappaport–Vassiliadis soy broth (RVS broth, Oxoid, Hamburg, Germany), incubated at 41.5 °C for 24 h. From these cultures, two selective solid media were inoculated: Xylose Lysine Deoxycholate (XLD) agar (Thermo Fischer Scientific, Erlangen, Germany) and Rambach agar (Thermo Fischer Scientific, Germany), the latter complementing XLD agar for isolating lactose-positive *Salmonella* and *Salmonella typhimurium*. Both XLD and Rambach agars were incubated at 37 °C and examined after 24 h. On XLD media, presumptive colonies of *Salmonella* appeared as red or pink with or without a black center, and on Rambach media, the colonies that were tested further were red and well differentiated. Confirmation tests included morphological (Gram staining), biochemical (urease reaction, lysine decarboxylase, indole test, H_2_S production), and serological identification (polyvalent antisera for flagellar (H) and somatic (O) antigens) of colonies grown on selective solid media.

#### 2.3.3. *Listeria* spp. Isolation Protocol

*Listeria* isolation followed the protocol specified in SR ISO EN 11290/1/2/2005 [49]. Samples were introduced into selective liquid enrichment media for primary enrichment (semi-Fraser broth) (Alpha Biosciences, Baltimore, MD, USA), then incubated at 30 °C for 24 h. Secondary enrichment was performed in Fraser broth (Alpha Biosciences, USA) with a regular concentration of selective agents (Fraser broth), incubated at 37 °C for 48 h. Cultures were streaked onto two selective solid media: Oxford agar (Oxoid, Germany), incubated aerobically at 37 °C for 24 h, and Palcam agar (Oxoid, Germany), incubated in microaerophilic conditions at 37 °C for 24 h. Characteristic colonies (gray or blue-gray with a surrounding black halo) were subjected to confirmation tests, including Gram staining, a catalase test, a hemolysis test, and a motility test.

#### 2.3.4. Bacterial DNA Extraction

The PCR technique used for confirmation began with bacterial DNA extraction, followed by amplification, migration, and result interpretation. DNA extraction followed a protocol described by Mihaiu et al. in 2014 [50]. Briefly, Eppendorf tubes (1.5 mL) containing 150 µL of 10% Chelex reagent (BioRad, Munchen, Germany) were placed under a microbiological hood (Microbiological Laminar Flow—Class II) under UV light for 15 min to eliminate potential contamination of the reagent or accidental contamination during handling. Using sterile microbiological loops, 1–2 characteristic colonies from positive selected plates (TBX for *E. coli*, Palcam for *Listeria*, XLD—for *Salmonella*) were transferred into the reagent. The extraction protocol included sequential exposure to 56 °C for 30 min and 92 °C for 5 min to lyse cell membranes and release genetic material. The final step involved centrifugation at 14,000 rpm, after which samples were stored at 4 °C until further analysis. DNA was extracted from the colonies identified as positive in the classical isolation protocol. Approximately 10–12 single colonies were processed with the Isolate II DNA kit (Bioline, London, UK) following the manufacturer’s instructions. DNA quantity and purity were assessed using a Nanodrop ND-1000 spectrophotometer (NanoDrop Technologies, Inc., Wilmington, DE, USA).

#### 2.3.5. PCR Confirmation of Pathogenic Strains

The amplification protocol involved placing the following into 200 µL Eppendorf tubes—12.5 µL of MyTaq (Bioline), 1 µL of Forward primer, 1 µL of Reverse primer, 4 µL of DNA, and 6.5 µL of PCR-grade water—resulting in a final volume of 25 µL. The primers used are described in Table 1. Amplification of the samples was carried out according to an optimized protocol in a thermocycler: denaturation, 1 cycle of 30 s at 95 °C; primer annealing, 30 cycles of 30 s at 56 °C; and final elongation, 1 cycle of 10 min at 72 °C. Sample migration in the gel was performed using an electrophoresis apparatus (CleverScientific, Rugby, UK) set to 100 W for one hour. The agarose gel used for the migration of amplified DNA samples had a concentration of 2.5%, and a GelGreen stain (Biotium, Fremont, CA, USA) was applied for visualization (1.7 µL for 35 mL gels and 7 µL for 170 mL gels).

All *E. coli* isolates underwent multiplex and/or single PCR analysis to detect genes associated with Shiga toxin-producing and extra-intestinal pathogenic *Escherichia coli*. The genes investigated for extra-intestinal pathogenic *Escherichia coli* included *cvaC* (colicin V), *KspII* (capsular polysialic acid virulence factor), *papC* (P fimbrial adhesin), and *fyuA* (yersiniabactin receptor for ferric yersiniabactin uptake). Additionally, we screened for genes encoding Shiga toxin types 1 and 2 (*Stx-1*, *Stx-2*), cytotoxic necrotizing factor (*Cnf1*), and *eaeA*, which is responsible for intimin-mediated attaching and effacing mechanisms.

#### 2.3.6. Data Analysis

Data gathered from aerobic colony counts (ACCs) and *Enterobacteriaceae* were presented as log CFU cm^−2^ and analyzed using mean ($\bar{x}$) values for comparison. Values that varied by less than 0.5 ($\bar{x}$) or 1.0 log CFU cm^−2^ (log N) were considered practically equivalent. The values of 0.5 or 1.0 log CFU/cm^2^ considered as thresholds for practical equivalence were addressed based on biological relevance and measurement variability. Bacterial counts can fluctuate due to sampling, environmental factors, and inherent biological variation, and the small differences (e.g., <0.5 log) that we detected we did not consider meaningful, as variations within 0.5 or 1.0 log CFU/cm^2^ do not significantly impact food safety risks or product quality. These factors were considered as random effects. Statistical analysis (ANOVA) was conducted with IBM SPSS Statistics 20 (IBM, Armonk, NY, USA), applying analysis of variance and Bonferroni corrections to examine differences in bacterial counts at the two processing stages examined (the post-evisceration and cooling stages).

## 3. Results

### 3.1. Results on the ACC and Enterobacteriaceae Loads

**Figure 1:**
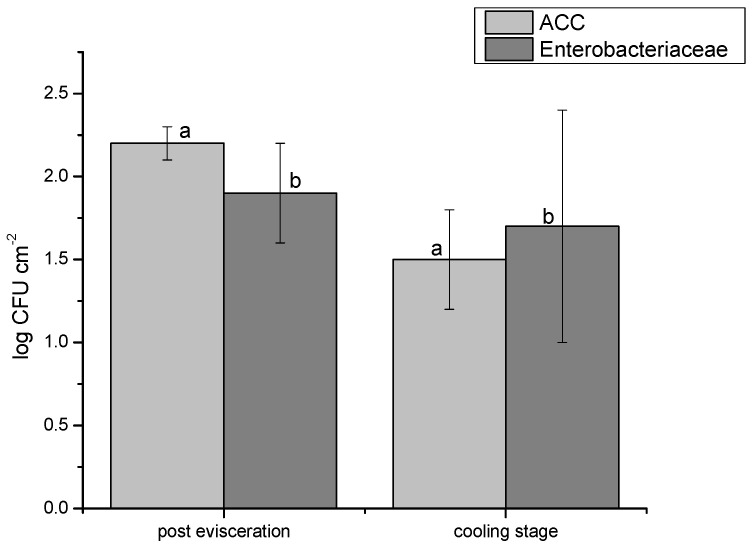
Graphical representation of aerobic colony counts (ACCs) and *Enterobacteriaceae* levels in the two main steps assessed (post-evisceration and cooling); a—ACC values (log CFU/cm^−2^) in the post-evisceration steps and cooling stage; b—*Enterobacteriaceae* levels (log CFU/cm^−2^) in the post-evisceration steps and cooling stage.

The assessment of aerobic colony counts (ACCs) in Romanian bovine slaughterhouses identified significant variations between samples taken immediately after evisceration and those collected during the cooling stage. Post-evisceration samples showed ACC values ranging from 2.1 to 2.8 log CFU cm^−2^, whereas cooling area samples exhibited statistically lower counts (*p* < 0.05), with values between 1.1 and 1.9 log CFU cm^−2^. The mean ACC value post-evisceration was 2.2 log CFU cm^−2^, which was markedly higher than the mean of 1.5 log CFU cm^−2^ recorded during the cooling phase (Figure 1), underscoring the importance of carcass hygiene controls in processing. Enterobacteriaceae levels also showed clear differences between these stages, with 84% of cooling area samples (*n* = 42) recording values below 1.0 log CFU cm^−2^. In contrast, post-evisceration counts ranged from 0.7 to 2.2 log CFU cm^−2^, with three samples exceeding 2.0 log CFU cm^−2^. The mean Enterobacteriaceae count for cooling area samples was 1.7 log CFU cm^−2^, lower than the 1.9 log CFU cm^−2^ mean recorded post-evisceration (Figure 1). These data reveal statistically significant differences (*p* < 0.05) between the two stages.

### 3.2. Results on the Prevalence of the Studied Bacteria in Fecal Samples

The prevalence of pathogenic bacteria from the studied genera varied among the samples. A total number of 110 *E. coli* strains were isolated from the fecal samples examined. All isolates were examined for the presence of 10 virulence genes commonly found in pathogenic *E. coli*. Potentially pathogenic *E. coli* strains, carrying one or more targeted virulence genes, were identified in 37.27% of the isolates (*n* = 41), while the remaining 62.72% (*n* = 69) tested negative.

Among the 41 isolates carrying virulence factors, the most frequently detected genes were *papC* and *fyuA*, each found in 9 isolates (21.95%). Other detected genes included *stx2* (19.5%; *n* = 8), *cnf*1 (19.5%; *n* = 8), *kpsII* (12.1%; *n* = 5), *cvaC* (9.7%; *n* = 4), *stx1* (9.7%; *n* = 4), and eaeA (4.8%; *n* = 2) (Table 2).

The tested isolates displayed 12 distinct patterns of virulence genes (Table 3). Two isolates contained four virulence genes, four isolates contained three virulence genes, eleven carried two, and the rest harbored only one. The three most common patterns involved isolates with a single virulence gene. Fortunately, carcass sampling yielded only 32 isolates. Molecular testing for virulence genes in these samples showed that only six were positive, all for the *papC* gene. Notably, the carcass samples positive for the *papC* gene originated from the same individual who also tested positive in fecal samples. This could suggest possible contamination during the slaughtering process.

Regarding the presence of *Salmonella* in the fecal samples, we found 14 positive samples, representing 28% (*n* = 50). The samples were also tested by the PCR method. Using PCR to discriminate the serotypes, four samples were confirmed as *S. typhimurium*, representing 28.57% of the positive samples, and one as *S. enteritidis*, representing 7.14% of the samples. Regarding the presence of *Listeria* spp. bacteria, only two of the samples were found to be positive and confirmed by PCR. None of them were found to be positive for *Listeria monocytogenes*.

### 3.3. Results on Carcass Sample Prevalence

Of the total carcass samples (*n* = 100), 5% (*n* = 5) tested positive for pathogenic *E. coli*, showing the presence of one or more virulence genes. Table 2 details the confirmed toxins, strain count, and serogroup. Two of the samples of pathogenic *E. coli* isolated from carcasses were from the same individuals that tested positive in the fecal samples examined. The strains were isolated in the post-evisceration step and also the cooling step. The other three strains that were identified were probably due to cross-contamination during processing or other methods of contamination due to lack of proper hygiene. None of the samples collected from carcasses showed positivity for *Salmonella* spp. or *Listeria* spp.

## 4. Discussion

The findings of our study underscore the significant risk of contamination with pathogenic microorganisms during beef slaughtering, particularly highlighting the importance of maintaining stringent hygiene practices after evisceration. Pathogens like *E. coli*, which are not always routinely monitored, continue to pose a notable risk for foodborne illnesses. One potential explanation for this issue is the variability in pathogen prevalence across regions and slaughtering practices. Our study found a 14% prevalence of *E. coli* in fecal samples, with serogroups O101 and O26 being particularly prevalent. A similar study conducted by Bonardi et al. (2014) [56] in a slaughterhouse in Northern Italy focused on fecal contamination with different types of pathogenic *E. coli*. In both studies, animals were brought from various sources: Italian farms and, in our study, local households reflecting Romania’s predominant cattle-raising practices. Bonardi et al. [50] detected a 13.1% prevalence of *E. coli* STEC strains in feces, similar to our 14% prevalence. Our findings showed the highest prevalence for serogroup O101 (seven and five samples, respectively), while Bonardi et al. [56] found O157 as the most prevalent. Differences were noted for serogroup O26, which appeared only in one of the samples in our study (14.28%), which is still a higher prevalence compared to 3.8% in Italy. Notably, O26 is the second most common serogroup in Europe after O157 in patients with foodborne illness, as reported by EFSA and ECDC in 2023 [57]. Studies in the U.S. have shown *E. coli* O157 in bovine feces with prevalence rates between 10% and 28% [58]. In 2022, the most frequently reported STEC serogroups in the EU/EEA were O157, O26, O103, O146, O145, and O91, collectively accounting for over 60% of confirmed cases, with a notable increase in the prevalence of O146 and O26, and a high association of O26 with severe cases [59]. In contrast, our study aimed to identify various serogroups, including O26, which, although not the primary cause of foodborne illness in Europe, remains a relevant pathogen with a prevalence of 37.2% in our positive samples.

This regional variation may result from differences in farming techniques, animal feed, or pathogen detection methods. Our study’s identification of *E. coli* O26, a significant pathogen in Europe, though less prevalent than O157, emphasizes the importance of expanding surveillance efforts to include multiple *E. coli* serogroups. Given the potential for regional differences in pathogen prevalence, a more comprehensive monitoring strategy is essential for effective risk assessment and food safety management. To mitigate the risk of *E. coli* contamination, it is essential to adopt more comprehensive monitoring protocols that include various serogroups beyond the commonly tested O157. Additionally, implementing improved hygiene measures during the evisceration and post-evisceration phases is critical, as pathogens like *E. coli* are easily transferred from fecal matter during these stages [60].

Regarding *Salmonella* spp., our study found a prevalence of 28% in fecal samples, with *S. typhimurium* and *S. enteritidis* being the most common strains detected. This prevalence is higher than that reported in some other studies, such as Bonardi et al. (2017) [61], who found no *Salmonella* in fecal samples. Fegan et al. (2004) [62] found a 6.8% prevalence of *Salmonella* in slaughterhouses, independent of the cattle-rearing system, further highlighting regional and methodological variability in detecting pathogens. Carcass *Salmonella* contamination may arise from fecal contamination during evisceration or from contact with animal hides during skinning, which can affect multiple pathogen types beyond *Salmonella* [63]. The variability in *Salmonella* prevalence across studies could stem from differences in slaughterhouse conditions, cattle-rearing practices, and sampling methods. While no *Salmonella* was found in carcass samples post-evisceration, its presence in feces prior to slaughter highlights the potential for cross-contamination during slaughtering. To reduce *Salmonella* risks, it is crucial to prevent fecal contamination during evisceration and avoid contact with contaminated animal hides during skinning. Ensuring thorough cleaning and sanitization of equipment and surfaces involved in these processes is essential.

As for *Listeria monocytogenes*, our study did not detect it in either fecal or carcass samples, though previous studies have reported varying prevalence rates. Similarly, research by Buncic et al. [64] in Yugoslavian slaughterhouses and retail locations identified a 19% prevalence for *L. monocytogenes*. Other studies reported lower prevalence rates, such as Zhao et al. [65] (9.7%) and Demaitre et al. [66] (46%). The differences observed in *L. monocytogenes* prevalence between studies could stem from variations in bovine feeding practices; silage-fed cattle tend to have higher *L. monocytogenes* incidence than grass- or hay-fed cattle. Although we did not observe *Listeria* contamination, its potential presence in other regions suggests that ongoing surveillance is necessary, particularly in environments with higher risks, such as those using silage in cattle diets.

Our study primarily focused on cattle, potentially limiting the scope of pathogen prevalence across different species. Additionally, the study only examined two abattoirs, which may not fully represent the broader range of slaughterhouse conditions across Romania. While samples were collected at two critical stages of processing, the study did not account for variations in other stages such as transport and holding periods before slaughter, which may also influence contamination levels. Future research could focus on expanding the sample size to include more abattoirs, from different regions and with varying processing capacities, to provide a more comprehensive understanding of pathogen prevalence across different types of livestock and slaughterhouse environments. Incorporating additional stages of meat processing, such as transport, holding, and packaging, would offer a more complete picture of contamination risks.

## 5. Conclusions

In conclusion, this study provides valuable insights into the microbiological risks in Romanian bovine slaughterhouses, highlighting the prevalence of *E. coli* and *Salmonella* in fecal samples, and the effectiveness of hygiene measures in reducing contamination during processing stages. Significant reductions in aerobic colony counts (ACCs) and Enterobacteriaceae levels between post-evisceration and cooling stages underscore the importance of proper hygiene protocols. About 37.27% of *E. coli* isolates from fecal samples carried one or more virulence genes, with *papC* and *fyuA* being the most frequently detected, emphasizing the need for stringent monitoring to prevent contamination. The detection of *E. coli* with virulence genes in both fecal and carcass samples, particularly from the same individual animal, suggests possible cross-contamination during processing, reinforcing the necessity for improved hygiene during evisceration and carcass handling. *Salmonella* was found in 28% of fecal samples, with *S. typhimurium* and *S. enteritidis* being the most common serotypes. However, this study’s limitations include the small sample size, especially from Abattoir B, and the focus on only two slaughterhouses, which may not fully represent broader industry trends. Additionally, the study did not consider contamination risks from stages such as transport and holding prior to slaughter. To build on these findings, future research should expand the sample size, include more diverse slaughterhouses, and explore other stages of meat processing, feeding practices, and the effectiveness of various intervention strategies to enhance food safety practices in the meat industry.

## Figures and Tables

**Table 1:** Specific primer sequences and targeted genes for pathogen confirmation.

Microorganism	Primer Sequence	Targeted Gene	Amplicon Size (pb)	Reference
*E. coli*	CTTTGACGGTAGTTCACTGGACTTC (F) GAAGACGTTATAGCCCAACATATTTTCAGG (R)	*eae*	166	[51]
	FCTTCGGTATCCTATTCCCGG (F) RGGATGCATCTCTGGTCATTG (R)	*Stx-1*	484	[52]
	CCATGACAACGGACAGCAGTT (F) CCTGTC AACTGAGCAGCACTTTG (R)	*Stx-2*	779	
	GGCGACAAATGCAGTATTGCTTGG GACGTTGGTTGCGGTAATTTTGGG	*Cnf1*	552	
	AACGCTATTCGCCAGCTTGC (F) TCTCCCCATACCGTACGCTA (R)	*ArpA*	400	
	ATGGTACCGGACGAACCAAC (F) TGCCGCCAGTACCAAAGACA (R)	*fhuA*	288	
	CAAACGTGAAGTGTCAGGAG (F) AATGCGTTCCTCAACCTGTG (R)	*YjaA*	211	
	GCGCATTTGCTGATACTGTTG (F) CATCCAGACGATAAGCATGAGCA (R)	*KspII*	272	
	GACGGCTGTACTGCAGGGTGTGGCG (F) ATATCCTTTCTGCAGGGATGCAATA (R)	*papC*	328	
	CACACACAAACGGGAGCTGTT (F) CACACACAAACGGGAGCTGTT (R)	*cvaC*	680	
*Salmonella* spp.	ATCGCTGACTTATGCAATCG (F) CGGGTTGCGTTATAGGTCTG (R)	*Omp* C	204	[53]
*S. typhimurium*	TTGTTCACTTTTTACCCCTGAA (F) CCCTGACAGCCGTTAGATATT (R)	*Spy*	401	
*S. enteritidis*	TGTGTTTTATCTGATGCAAGAGG (F) TGAACTACGTTCGTTCTTCTGG (R)	*Sdf*I	304	
*Listeria* spp.	GCTGAAGAGATTGCGAAAGAAG (F) CAAAGAAACCTTGGATTTGCGG (R)	*Prs*	370	[54]
*L.* *monocytogenes*	TCATCGACGGCAACCTCGG (F) TGAGCAACGTATCCTCCAGAGT (R)	*prf*A	217	[55]

**Table 2:** The occurrence rate of the tested genes among the isolated *E. coli* strains.

Targeted Gene	No. of Positive Samples/%
*papC*	9/21.95
*fyuA*	9/21.95
*stx2*	8/19.5
*cnf*1	8/19.5
*kpsII*	5/12.1
*cvaC*	4/9.7
*stx1*	4/9.7
*eaeA*	2/4.8
*ArpA*	0
*YjaA*	0

**Table 3:** Characterization (virulence genes and serogoups) of *E. coli* isolates (*n* = 110) collected from fecal samples in Romanian slaughterhouses.

Virulence Gene Detected	No. of Positive Strains	% of Positive Samples (*n* = 110)	Serogroup
*papC*	1	0.9	O101 ((K99)-F41-positive)
*fyuA*	4	3.63	O101 ((K99)-F41-positive)
*Stx-2*	2	1.8	O26, O101 ((K99)-F41-positive)
*Cnf1*, *Stx-2*	4	3.63	O26, O101 ((K99)-F41-positive)
*Stx1*, *Stx-2*	2	1.8	O101 ((K99)-F41-positive)
*eaeA*, *Stx-1*	2	1.8	O101 ((K99)-F41-positive)
*Cnf1*, *kpsII*	1	0.9	O26, O101 ((K99)-F41-positive)
*papC*, *fyuA*	2	1.8	O101 ((K99)-F41-positive)
*cvaC*, *papC*, *kpsII*,	2	1.8	O26, O101 ((K99)-F41-positive)
*papC*, *fyuA*, *cnf1*	2	1.8	O101 ((K99)-F41-positive)
*cvaC*, *papC*, *kpsII*, *cnf1*	1	0.9	O101 ((K99)-F41-positive)
*fyuA*, *cvaC*, *papC*, *kpsII*	1	0.9	O26, O101 ((K99)-F41-positive)

## Data Availability

Data are unavailable due to privacy or ethical restrictions.
